# Virtual Clinics in Cardiology: Do They Provide Equivalent Care and Reduce Travel?

**DOI:** 10.3390/jcm14207363

**Published:** 2025-10-17

**Authors:** Matthew Farrier, Brian Wood, Zoubaida Yahia, Martin Farrier

**Affiliations:** Wrightington, Wigan and Leigh Teaching Hospitals NHS Foundation Trust, Wigan WN1 2NN, UK; mtfarrier@gmail.com (M.F.); brian.wood@wwl.nhs.uk (B.W.); zoubaida.yahia@wwl.nhs.uk (Z.Y.)

**Keywords:** telemedicine, investigation, travel

## Abstract

**Objective:** To evaluate whether virtual clinic appointments in cardiology are equivalent to face-to-face appointments in terms of investigations as a consequence of the appointment and a reduction in travel for the whole care episode. Design: Retrospective observational cohort study of 9445 patients. Setting: Wrightington, Wigan and Leigh Teaching Hospitals NHS Foundation Trust, a medium-sized NHS trust in the north-west of England. Participants: 9445 patients referred for new cardiology appointments between 2023 and 2025. **Methods:** Data were extracted from electronic records and test ordering systems and appointments with corresponding investigations were retrieved. The data was validated using random samples, and the extraction was modified until accuracy was achieved. Principle component analysis was used to compare groups, and Welch *t*-test was used to statistically analyse the results. Distance travelled was calculated using postcodes and the number of visits was calculated using investigations conducted on separate days. **Results:** Patients who had virtual appointments showed no statistical difference in the number of investigations or visits for investigations. The care provided via virtual and face-to-face appointments was found to be comparable in terms of clinical effectiveness and quality of care. The distance travelled for both types of appointment is therefore not different, but if the initial appointment is taken into consideration where there was no travel for the virtual appointment patients, then the reduction in miles travelled is 5002 km, resulting in a carbon saving of 784 kgCO_2_eq. **Conclusions:** Virtual Clinics in Cardiology offer an equitable service but only a small reduction in travel.

## 1. Introduction

Virtual clinics have been increasingly used as part of modern healthcare. There was a significant increase in the use of virtual clinics around the time of COVID, and they are now established as a normal part of care. The NHS entered into a contractual arrangement with Attend Anywhere to provide video software that supports appointments. However, the use of telephone to conduct appointments remains more popular than the use of such software. By whatever means they are provided, virtual appointments are widely accepted as being convenient [1] and are popular with patients [2,3]. Their outcomes have been less well established.

When virtual appointments were first provided, the benefit was not based on a reduction in travel time or convenience, but aimed to improve infection control measures [4]. Since then, virtual appointments have continued to be used for convenience. There was an assumption that they also led to less travel and thereby reduced carbon emissions from the transport vehicles used. Travel makes up an important part of the carbon footprint of healthcare, and reductions in travel could potentially help reduce the carbon footprint of healthcare. While work has been done to establish that they actually lead to less travel for the initial appointment [5,6,7], some have hypothesised that because doctors are less confident about the information in a virtual appointment, they may compensate by ordering more investigations or increased follow-up from the initial virtual appointment [8]. It is suggested that the inability to examine patients will lead to more investigations. Offiah et al. indicated that there were fewer investigations after virtual appointments, but the study was conducted during COVID and could not account for selection bias [9]. During the COVID period, selection for virtual appointments was mostly linked to timing and isolation, whereas for our work in the time after COVID, selection is made by our appointments team. Were virtual appointments to lead to more investigations, travelling distances might actually be higher and the appointments would neither save time nor reduce the carbon footprint of healthcare. This work evaluates whether virtual clinic appointments lead to more investigations and thus increased total travel distance and whether the care provided by virtual appointments is equitable with face-to-face visits. Previous published work has indicated that virtual appointments are equitable with face-to-face appointments in terms of value and information [10]. Further work is needed to establish whether investigations for both groups are comparable in effectiveness and quality of care and is addressed in this study.

## 2. Materials and Methods

### 2.1. Study Design

We conducted a retrospective observational cohort study of 9445 patients who attended either virtual (4672 patients) or face-to-face (4773 patients) cardiac clinic appointments. The study was conducted in a single centre (Wrightington, Wigan and Leigh Teaching Hospitals NHS Foundation Trust, a single medium-sized NHS trust in the north of England), and involved a single cardiology department. The appointments were allocated according to space in the clinic rather than patient preference. The booking team indicated that no selection criteria were applied to the appointment allocation. Patients were taken from the referral list and allocated the first new appointment available on the appointments list. This list contained both virtual and face-to-face appointments, and both were treated equally. We retrospectively gathered data on the number of tests ordered and the distance travelled for the cohorts. We also gathered data on the demographics of the patients to ensure that the 2 groups were matched.

Ordering of the tests was completed at the time of the appointment by the cardiologist. All orders are electronic and can be tracked and linked to the investigations completed. Investigations are reported electronically, allowing for the timing of the tests to be retrieved from the data retrospectively. The demographic details of the patient are stored electronically and checked at the time of attendance. These details include a post code, which was used to calculate the distance travelled.

### 2.2. Setting

The participants were seen in either face-to-face clinic or virtual clinic appointments at the outpatient clinics of the Trust. Virtual appointments could be conducted via video or telephone. Appointment type (virtual or face-to-face) is routinely recorded, but whether the consultation used telephone or video software to complete the consultation is not recorded and cannot be determined. Discussions with the consultant cardiologists indicates that they used both, but that telephone is the more common medium.

### 2.3. Time Scale of Data

The study included data from 9445 patients seen between January 2023 and June 2025. Data from earlier years was accessed and is available. However, that time was influenced by the COVID pandemic and movement restrictions. The data from this time was not included as there was a potential for bias around travel. The last travel restriction in the UK was lifted in March 2022 [11] and there were no travel restrictions by 2023, with society having returned to normal behaviours. The study was restricted to a stable time period where restrictions were absent and the clinics ran without any changes to the format or the booking arrangements. Data extraction was completed during 2025. Initial data extractions were completed early in 2025 to allow for validation work, and the final data extraction was completed in July 2025.

### 2.4. Participants

Participants were allocated by the booking teams to either virtual or face-to-face clinic appointments from a shared pool of new referrals from the patients’ general practitioners, emergency attendance at the Emergency Care Centre, or inpatient referrals. The booking teams indicated that they did not select patients for slots in clinics, but that patients were allocated a slot according to availability. This was expected to lead to a random distribution of patients between virtual and face-to-face clinics. Further testing of that assumption will be conducted using demographics of patients.

The participants were analysed according to the 2 groups (virtual and face-to-face) for the subsequent investigations ordered as a consequence of the initial appointment.

The study was conducted retrospectively, and consequently, consent could not be obtained from the patients for their inclusion in the data. Individual patient data was not identifiable.

### 2.5. Data Source

Data within the Trust is collected as part of the process of booking clinical appointments with patients. The booking clerks use the Patient Administration System (PAS) to allocate and book patients. Consultants use the Trust Electronic Patient Record System (based on Altera Sunrise) to record the consultation and order tests. Most tests results are entered directly into the EPR system. Cardiac-specific tests such as ECG are recorded within PRISM, which is an electronic system specific to cardiac investigations. Data from all these systems could be accessed and extracted for analysis purposes in this study. Data extraction was automated and built as a query sequence, with data queries mapped to a single outcome dataset. Validation was performed during the initial process of building queries in order to ensure that they were correct and then to validate the data within the final dataset. Where differences were found, the data queries were altered until there were no data discrepancies. Validation of the final dataset was completed with 50 randomly selected cases.

All tests ordered by the consultant cardiologist who saw the participant were assumed to be related to clinic appointments and counted. Distance travelled was calculated using the postcodes patients had provided as their current living address at the time of the appointment being booked. If 2 tests were conducted on the same day, they were assumed to have been done in a single visit, so that travel was only counted once for the 2 tests.

## 3. Results

### 3.1. Allocation of Appointments

**Figure 1:**
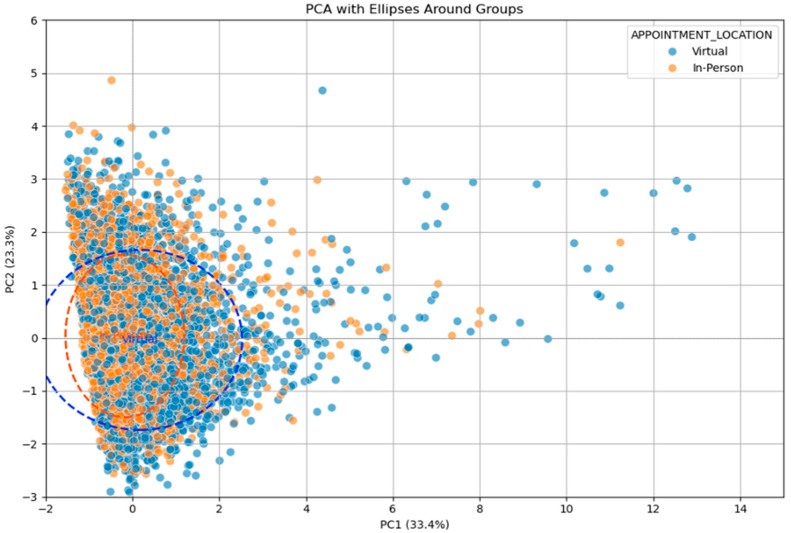
PCA analysis of distance travelled by virtual (blue) and in-person (orange) patients, with ellipses plotted around the groupings.

The allocation of patients between virtual and face-to-face appointments was considered to be non-selective by the booking team. However, logically, people who have large distances to travel are likely to ask for virtual appointments more than the ones who live next to the hospital. While there is no policy or attempt to implement this strategically, we gathered data on the 2 groups and performed Principal component analysis (PCA) to examine the similarity of the groups. The booking clerks indicated that there was no process for selecting patients between virtual and face-to-face appointments. The PCA, however, indicates that there is a difference between the 2 groups. The virtual group of patients is more likely to travel further (Figure 1). This is a small effect, however, and there is a high degree of similarity between patients.

**Figure 2:**
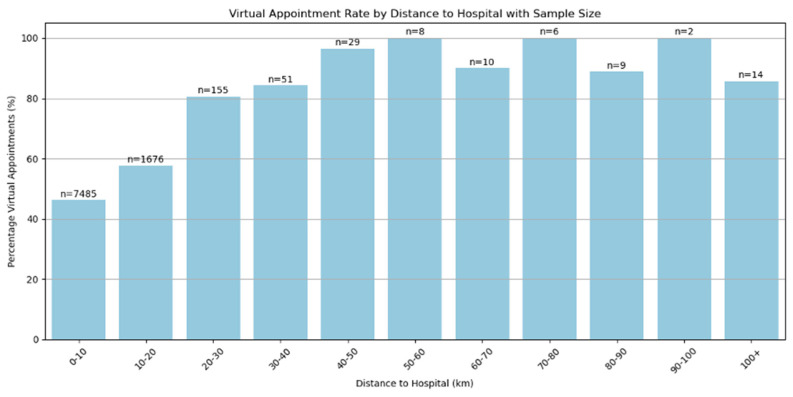
Percentage of patients with virtual appointments compared to the distance travelled.

When travelling distances are small, there is little difference in the allocation of appointments between virtual and face-to-face (Figure 2). Only when distances exceed 20 km does the difference become important. However, there were only 284 patients from the total study population of 9445 (3.1%) who travelled over 20 km for an appointment.

### 3.2. Principal Component Analysis

Principal component analysis (PCA) is used to examine the relationship between multiple factors within the 2 datasets. Using PCA allows for complex relationships between factors to be visualised, such that 2 groups of patients can be compared. Figure 1 indicates that virtual and face-to-face patients are very similar in their characteristics. The virtual patients (blue) travelled further, and this is illustrated by the greater variation of the blue plotted ellipsoid compared to the orange plotted ellipsoid (blue and orange dotted lines). However, the overlap between the ellipsoids is significant, with the similarities thereby demonstrated. PCA indicates that the virtual and face-to-face groups of patients are very similar and that there is little selection bias between the 2 groups.

### 3.3. Outliers for Distance Travelled

**Figure 3:**
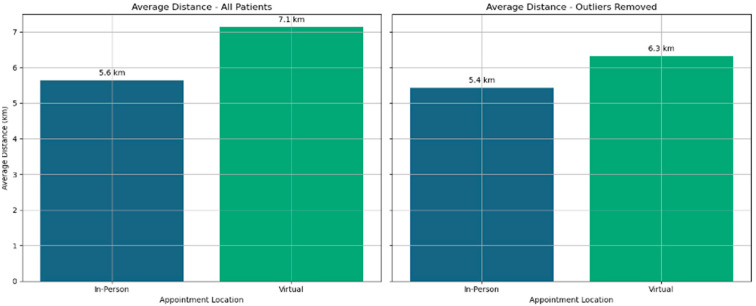
Average distance travelled by all patients and by patients from whom outlier distances are removed.

Distance travelled by the patients was typically under 20 km. Just a very small number of patients travelled large distances, and they likely represented a very different population from the main cohort of patients. Possibly, they were no longer living in the area but attending a review after an emergency appointment. Validation work could not establish the reasons for the larger travelling distance, as it was rarely recorded. Calculation of the distance travelled by patients was completed for all patients. To reduce the effect of extreme outliers for distance travelled, these extreme outliers were excluded and average distances travelled were also calculated (Figure 3). The distance for patients who attended virtually was higher in both calculations.

**Figure 4:**
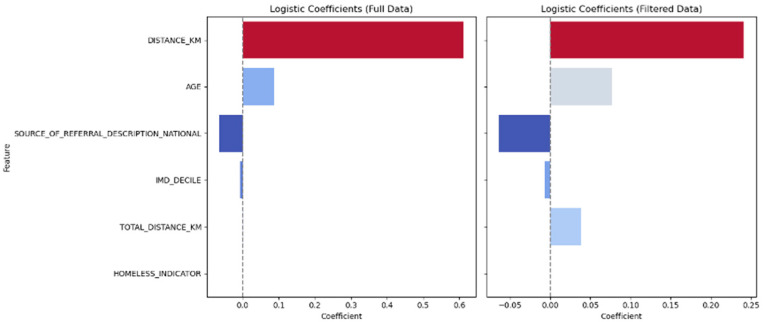
Comparison of patient features between virtual and face-to-face appointment types.

Further analysis of the differences between virtual and face-to-face appointments shows that there is a significant difference between the 2 groups for distance travelled but not for age, type of referral, frailty, and homelessness. This remains true after the removal of large distance outliers (Figure 4).

### 3.4. Number of Investigations Performed

**Figure 5:**
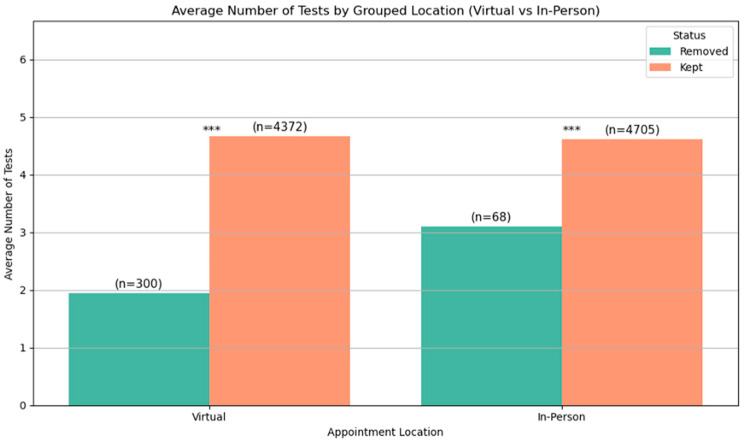
Average number of investigations per patient expressed for virtual and in-patient attendance. Outliers for distance travelled are excluded (orange), but are included (green) for comparison. *** indicates *p* < 0.05.

The number of investigations performed after each appointment type was accessed and is represented in Figure 5. The average number of investigations performed was the same for both virtual and face-to-face patients. Analysis using Welch’s *t*-test demonstrates statistical significance of less than 0.05 (*p* < 0.05). Welch’s *t*-test was used as it does not require equal sample sizes. Patients who were outliers for distance travelled were excluded from the analysis, but their data is represented for comparison.

### 3.5. Number of Visits

**Figure 6:**
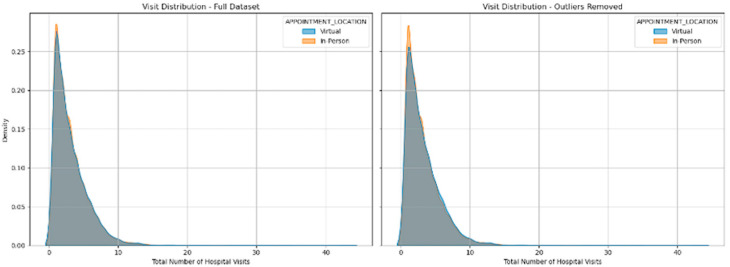
Frequency distribution of the number of tests performed for virtual and in-person appointments.

**Figure 7:**
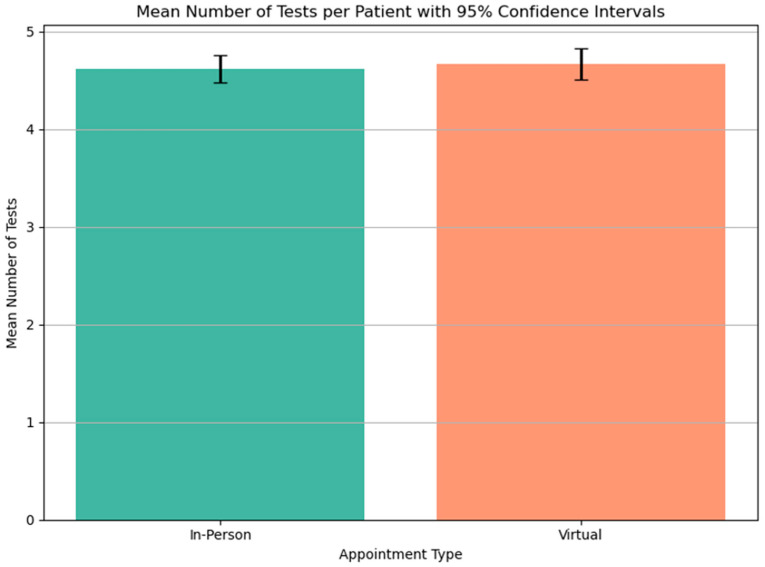
Mean number of tests per patient for virtual and in-patient attendances.

The number of visits for investigations was derived from the total number of investigations performed and corrected for investigations that were conducted on the same day. It was common for an ECG to be performed at the same time as another investigation, and so the patients did not need to travel simply to have an ECG done when they were attending for another test on the same day. The frequency distribution of the number of visits for each group is plotted in Figure 6. There was no significant difference in the number of visits made by the 2 groups (Figure 7).

### 3.6. Distance Travelled

**Figure 8:**
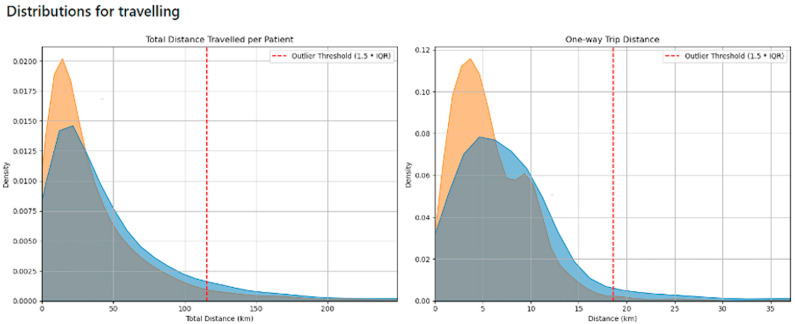
Distribution of distance travelled for virtual (blue) and in-person (orange) attendance. Total distance for all investigations and one-way distance for a single investigation are shown.

The distance travelled for the tests performed is shown in Figure 8. The distance travelled is marginally greater for the patients seen in virtual clinics because of the longer distance (6.4 vs. 5.3 km) they travel to have the investigations performed. The data presented only considers the distance travelled for investigations and does not include the initial consultation, where there is no travel for virtual clinic attendance.

The total distance travelled by patients in the virtual clinic group, when considering the initial appointment and the investigations, is less than that of the face-to-face group because of the initial visit. The total reduction in distance travelled amounts to 5002 km.

## 4. Discussion

This study examines the distances travelled by patients attending virtual cardiac appointments in comparison to those attending face-to-face cardiac appointments. We include 9445 patients split between the 2 groups.

It was initially considered that patients were randomly distributed between virtual and face-to-face appointments because of the way appointments are allocated by the booking clerks. However, the analysis shows that there is some bias towards patients who live further away. It is likely that the booking clerks do not introduce this bias; rather, patients who make contact with the cardiac team and ask for a virtual appointment are then allocated such an appointment. This is a small effect, but because of the patients who live far away, it is evident in the PCA.

Other factors that might be expected to have an effect on appointment selection did not show a significant effect. Patients who are old or frail might be more likely to have virtual appointments in order to save them from a difficult journey, but this is not evidenced within our data. Homelessness might also make attendance at a virtual appointment more difficult, but again there is no evidence from our data to support this. It is possible that patients who are homeless are simply unable to access virtual services without owning a smartphone, but this cannot be evidenced within this data.

The number of visits made for investigation by patients after an initial cardiology appointment is the same whether they are virtual or face-to-face appointments. There is no increase in the number of investigations because the clinician did not have face-to-face interaction with the patient. There has been a suggestion that investigations are used as a way of examining patients who have not been examined at the initial appointment, but that is not evidenced here. Other studies have suggested that there may be a reduction in the number of investigations carried out, but the largest of these indicated greater follow-up and included many different specialities [8,9].

Consequently, the distances travelled by both groups for investigation are shown to be the same. There is an overall reduction in distance travelled by the patients who are initially seen in virtual clinic appointments. They will have 1 fewer visit as a consequence of the initial virtual visit, and that visit will require no travel. The average number of visits made for investigations was between 3 and 4 for both the virtual and face-to-face groups. The reduction in travel for the virtual appointments was around 25%, amounting to a reduction of 5002 km and a carbon saving of 784 kg CO_2_eq (assuming typical car engine types, all cars upper medium and typical public transport usage). This is a finding not previously evidenced and allows for an assessment of the benefits of virtual appointments as part of the reduction of the carbon impact of healthcare.

### Limitations

This study is a single-centre study and looks only at one group of patients. It cannot be assumed that the patterns of assessment in one organisation will be replicated in other organisations, in particular in other countries. The number of investigations conducted by cardiologists is in part dependent on the system that they work in, and the number of visits is in part determined by how that system works.

Data extraction and analysis are manually validated, but only for a relatively small sample of patients. However, the automation of data extraction allows for a large sample size. Entry of data at the point of testing is assumed to be accurate and cannot be validated. The mechanism by which tests are ordered and results are recorded against the electronic order makes data entry likely to be accurate.

Data collection on virtual attendance types (telephone and video) is not available and may have an effect on the behaviour of clinicians in terms of decision making and ordering of tests.

## 5. Conclusions

This study looks at virtual care within cardiac clinics. While clinics of other disciplines may have other types of clinical work, the nature of decision making is likely to be similar. The process of history taking and decision making will follow similar patterns, and as such, the study is likely to reflect clinical decision making rather than the specific nature of cardiology. The equitability of care within other clinic types and in other settings is likely to follow similar patterns. However, further work would be necessary to establish how generalisable the results are to other specialities.

Virtual cardiac appointments are well received by patients [2,3]. They offer an alternative to attending a clinic face-to-face. This study indicates that they provide care that is comparable in clinical effectiveness and quality to patients and do not lead to additional investigations. However, the initial appointment is only 1 visit out of a sequence. The process of investigation requires the patient to attend between 3 and 4 times in-person. The reduction in travelling is therefore relatively small as a component of the total travel for a cardiology assessment. The process of investigation could be coordinated and might have a greater impact on the reduction in travel than the initial type of appointment. Were all the investigations to be completed in a single attendance, the reduction in travel would be greater than the reduction achieved by the use of a virtual appointment. The combined effect of a virtual appointment with coordinated investigation could ultimately limit travel to a single trip. Some visits might further be replaced by the use of remote monitoring for devices where this is possible [12].

## Data Availability

Data is available by reasonable request by email from the corresponding author, Martin Farrier, at martin.farrier@wwl.nhs.uk.
